# Ischemic Stroke in a Young Man: Unraveling the Domain of Myeloproliferative Disorders

**DOI:** 10.7759/cureus.16495

**Published:** 2021-07-19

**Authors:** Muhammad Ammar B Hamid, Aasim S Sehbai, Shahan Tariq

**Affiliations:** 1 Hematology and Oncology, Alabama Cancer Care, Anniston, USA

**Keywords:** stroke, myeloproliferative disorder, essential thrombocythemia, mthfr mutation, stroke in young adults

## Abstract

Ischemic stroke is a rare phenomenon in young adults. A complete workup for hypercoagulable and myeloproliferative disorders is a cornerstone of evaluation. Essential thrombocytosis is a chronic myeloproliferative disorder that primarily involves platelets. It may remain undiagnosed in patients and subsequently present in the form of ischemic stroke. The management of this disorder is complex and often involves cytoreduction therapies. The initiation of these drugs in such patients may lead to unnecessary adverse effects and complications. This case report is an attempt to highlight an underappreciated cause of stroke when assessing young individuals.

## Introduction

Thrombocythemia often develops as a secondary response in reaction to a number of states including chronic inflammation, malignancy, infection, iron deficiency, trauma, or surgery, and is mostly diagnosed as an incidental finding. Although very infrequent, it may occur due to an underlying process related to cellular replication and proliferation. These uncommon conditions are classified under the heading of myeloproliferative disorders (MPDs) as essential thrombocythemia (ET), chronic myeloid leukemia (CML), scarring agnogenic myeloid metaplasia (AMM), and polycythemia vera (PV). Furthermore, mutations in the thrombopoietin gene can result in the occasional occurrence of familial thrombocythemia [1]. Through this case, we have attempted to highlight an uncommon cause of ischemic stroke in young individuals.

## Case presentation

**Figure 1:**
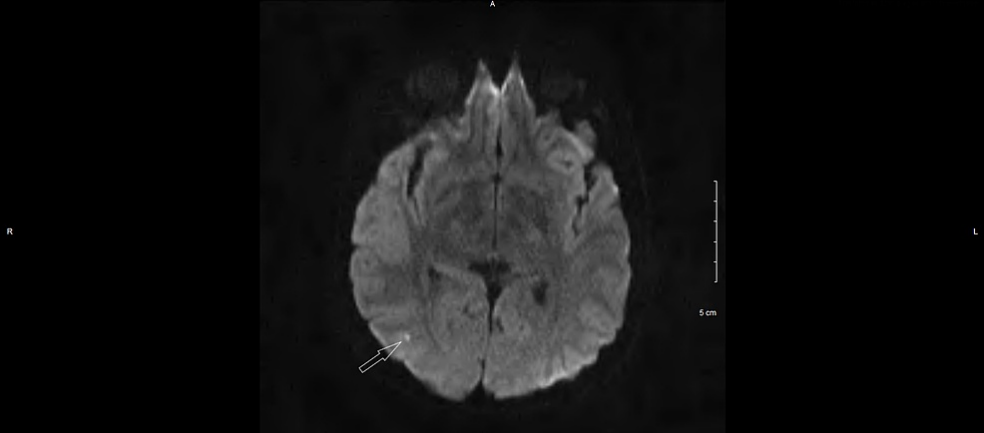
MRI of the brain showing a hyperintense lesion in the right parietal-occipital region

A 25-year-old man presented to the hematology/oncology clinic for a follow-up appointment. One week ago, the patient was admitted to the hospital with mild confusion and sudden onset weakness of the left side of his body. Motor strength was 2/5 in both the left upper and lower limbs. Babinski reflex could be appreciated in the left foot. An MRI of the brain (Figure 1) revealed an infarct lesion in the right parietal-occipital region consistent with a diagnosis of ischemic stroke. He was subsequently treated with tissue plasminogen activator (tPA) and later started on aspirin along with clopidogrel. Additional workup revealed an extremely high platelet count of 1030 x 10^9^/L. The rest of the cell counts were normal. Due to the unusual nature of this presentation in a young individual, a detailed workup for hypercoagulable and myeloproliferative disorders was initiated. The patient’s stroke symptoms improved over the next five days and he was eventually discharged.

He complained of a mild headache, fatigue, and blurry vision since being discharged. He was vitally stable with cardiovascular and respiratory examination being normal. The abdomen was soft, non-tender, bowel sounds were positive and no evidence of hepato-splenomegaly was found. His labs revealed hemoglobin (Hb) of 14.5 g/dL, white blood cell (WBC) count of 10.7 x 10^9^/L, and platelet count of 952 x 10^9^/L. His serum iron, total iron-binding capacity (TIBC), Vitamin B12, folate, and homocysteine levels were within the physiological range. Janus kinase 2 (JAK2) and methylenetetrahydrofolate reductase (MTHFR) heterozygous A1298C mutations were positive. The rest of the investigations including prothrombin time/partial thromboplastin time (PT/PTT), protein C, protein S, factor V, prothrombin gene mutation, anti-nuclear antibody (ANA), human immunodeficiency virus serology (HIV), rapid plasma reagent (RPR), lupus anticoagulant, anti-cardiolipin, and β-2 microglobulin antibodies were negative. Abdominal and duplex ultrasound of lower extremities were unremarkable. His past surgical history was positive for a 5.5 cm lipoma removal from his right shoulder. His past medical history did not reveal any significant findings. There was no history of any vitamin/mineral deficiency. No history of similar diseases in the family could be illustrated. He is a non-smoker, non-addict with no known drug allergies. He drinks alcohol socially.

A bone marrow biopsy showed hypercellularity and atypical megakaryocytic hyperplasia consistent with the diagnosis of essential thrombocytosis. Given his established history of thrombosis, he was advised to continue aspirin. Due to his young age, cytoreduction therapy with pegylated interferon-alpha (PEG-IFN-α) is being started at a low dose of 45 μg weekly and will be increased as required. From next week, hydroxyurea would be added to help while the effects of interferon kick in. He will be monitored with regular follow-up every four weeks.

## Discussion

Out of all the myeloproliferative disorders, CML is the only one characterized by a specific mutation in the BCR-ABL arrangement. The rest of the MPDs are known as BCR-ABL negative. The presence of persistent non-reactive thrombocythemia usually leads to the diagnosis of ET. The main features observed in ET are a significant rise in the number of platelets and megakaryocytes. The definite origin of ET can perhaps be associated with several underlying factors in various patient subsets. However, its pathophysiology apparently seems to be related to an acquired interference in the signaling pathway involving thrombopoiesis. JAK2 mutation is a guanine to thymine somatic mutation at nucleotide 1849 in exon 14 and occurs when a gain of function substitute replaces valine with phenylalanine at position 617. Genetic mutation for JAK2 has been found in a considerable number of patients suffering from either one of the three non-BCR-ABL MPDs i.e. PV, AMM, and ET [2,3], thereby essentially narrowing down the differentials by ruling out CML. Out of these, 23% to 57% of the patients having ET were also reported to have a mutation in JAK2 [4].

Differentiating between reactive and clonal thrombocythemia requires quite a number of investigations to be done which, no matter how laborious, are decisive in narrowing down the definite diagnosis whilst ruling out others as well as crucial in regard to employing the correct treatment protocols.

Although a large proportion of patients remain asymptomatic for a considerable duration, nearly 40% to almost 90% of patients suffering from ET show symptoms such as hemorrhage, thrombosis, and/or vasomotor disturbance when they ultimately present [5]. Thrombosis is three times more likely to occur in the arterial vasculature compared to the venous bed [6]. Around 30% to 40% of patients present with platelet-mediated vasomotor symptoms including lightheadedness, headaches, migraine-like symptoms accompanied by visual disturbances which are transient in nature, focal neurological deficits, and syncope [7,8].

In a study, the risk of thrombotic events such as stroke, myocardial infarction, deep vein thrombosis, transient ischemic attacks, pulmonary embolism, retinal artery/vein occlusion, thrombosis of portal/hepatic veins, and limb/digital ischemia in those having ET was calculated. It was found to be about 6.6% per patient annually compared to the control population, which had a risk of only 1.2% per patient annually [9]. JAK2-V617F mutation is present in approximately 50% to 60% of ET cases. It doubles the risk of thrombosis along with hypertension and smoking being substantial risk factors [10].

A retrospective chart review study involving patients having rare MTHFR polymorphisms concluded that there exists an increased risk of venous thromboembolism (VTE) associated with heterozygous/homozygous MTHFR variants. Meanwhile, hyperhomocysteinemia does not have a significant correlation with MTHFR polymorphisms. Therefore, assessment of VTE risk can be better estimated using genotyping of MTHFR [11]. Furthermore, the co-existence of ET and MTHFR mutation with hyperhomocysteinemia has been observed to result in an exponential increase with regards to the risk of occurrence of an ischemic phenomenon [12]. Our patient was a unique case having a mutation in MTHFR but normal homocysteine levels, an extremely rare entity.

Patients suffering from ET who have had an ischemic stroke are at higher risk for re-thrombosis and thus, they are additionally managed with cytoreductive agents along with antithrombotic medication. Given the abnormal production of platelets, there occurs only a sub-optimal suppression of platelets using the standard and recommended regimen of aspirin. Hence, to achieve ideal results, low-dose aspirin should be administered twice daily in an arterial thrombotic disease where the following risk factors are present: age of 60 and above, JAK2 mutation, and cardiovascular risk factors [13].

Treatment of ET revolves around the estimation of the relative risk of thrombosis and further transformation of disease. The risk of ET transforming to leukemia or other MPDs has been observed to increase over time [14]. Those above 60 years of age or having past history of thrombotic episodes are considered as high risk and should receive treatment with hydroxyurea, a cytoreductive agent. With regards to the treatment of thrombocythemia in ET, several RCTs have established the efficacy of hydroxyurea both when administered in comparison to a control as well as in combination therapy either with aspirin alone or anagrelide alongside [15,16]. IFN-α is also considered to be a reliable treatment, especially in pregnant or those who cannot tolerate hydroxyurea. IFN-α has the added benefit of resulting in a complete molecular response (CMR) where the JAK2-V617F becomes undetectable, although in fewer patients, alongside a complete hematologic response (CHR). Genotypic background may determine the ultimate clinical and molecular response to PEG-IFN-α; however, it is worthy of mention that patients who did not have other mutations outside the JAK2 signal transducer and transcription activator pathway were more likely to achieve CMR [17].

Extreme thrombocytosis is defined as a platelet count of more than or equal to 1,000 × 10^9^/L and such states are rarely observed. A retrospective study involving 535 patients having extreme thrombocytosis showed that only 2% were suffering from ET [18]. The initial presentation of our patient with ischemic stroke at the age of 25 and his subsequent diagnosis of ET along with a JAK2 mutation is no less than a rare occurrence. Hence, physicians should be mindful of the fact that there may exist underlying genetic predisposition in a patient of stroke or vice versa.

## Conclusions

It is important to consider essential thrombocytosis as a potential cause of ischemic stroke when dealing with a young adult. Although there is a need for further studies in order to estimate and increase the effectiveness and safety of all available treatment modalities, it must ultimately be intended for coping with all known modifiable risk factors and thereby, reduce them. This objective becomes of utmost importance when the patient is young, such as ours, who potentially has decades to deal with the side effects of treatment as well as accumulate additional risk factors.
